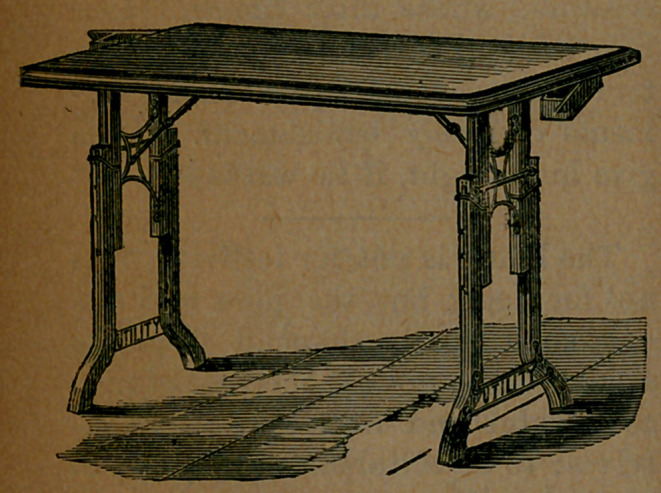# The “Utility”

**Published:** 1875-04

**Authors:** 


### THE "UTILITY."

We spoke last month of the neat and handy "Utility Adjustable Table," sold by Lambie, Sargent, & Co., No. 793 Broadway. We ordered one of the $10 style sent home for the ladies to experiment with. Its appearance excited a good deal of admiration, for it is a very graceful and showy piece of furniture. When put in practical daily use, its numerous advantages quickly won all hearts.

Here is a view of the table with its legs folded one upon the other. In this condition it is quite thin, and can be placed against the wall without taking up any useful space in the room. When a table is required for any purpose, this can be adjusted in a few seconds, at any desirable height, from twenty-two and a half to twenty-eight and a half inches. The lower height makes it very convenient for children, and for ladies who use it as a work-table. The last view shows it standing, with its little swinging drawer at each end.

One of its great charms is that it is always at hand and always ready. The other tables are certain to be burdened with books or other articles for which there seems to be no more convenient place. To clear off these, if you chance to want an empty table, is something of a task. But your "Utility" is at hand; and, in an instant, you drop the legs, straighten the braces, set the top—by the mere act of lifting it—at the height preferred, and your table is ready. Moreover, it is light and strong. When ready for use it stands firm and immovable. It can not warp, for it is made of many layers of wood, the grain of which runs in different directions, so that great strength and lightness is secured. It is a great favorite with the women, and is almost invaluable to persons who have writing to do. It makes as useful and acceptable a present as man, woman, or child, can receive. The manufacturers send illustrated circulars to all who apply for them, and may be depended upon for promptness and fair dealing.

---

Mr. Joel McComber wishes us to remind all who are seeking for his goods of dealers in boots and shoes, that all his patent goods are stamped with his name and the date of the patent. The boots and shoes are patented, as well as the lasts upon which they are made; and it is a felony to stamp goods not made on his lasts with his name.

## Figures and Tables

**Figure f1:**
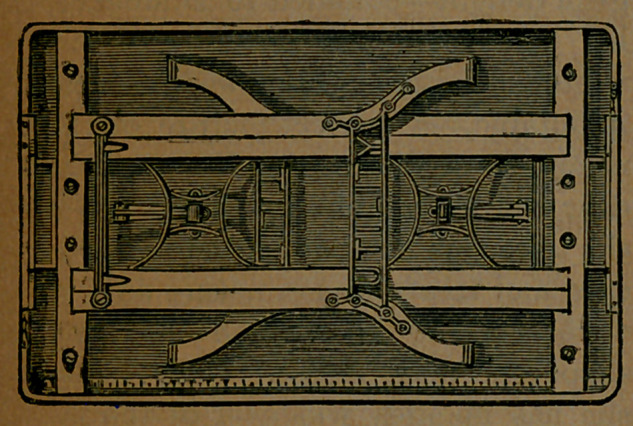


**Figure f2:**